# Management of malaria in newborns: a systematic review

**DOI:** 10.1186/s12879-025-12230-5

**Published:** 2025-12-05

**Authors:** Ayten Sultanli, Julia Selena Beck, Marlene Kremp, Hannah Sondermann, Maria Bonitz, Yabo Josiane Honkpehedji, Jonathan Remppis, Ayola Akim Adegnika, Meral Esen, Sabine Bélard

**Affiliations:** 1 Institute of Tropical Medicine, University of Tübingen, Wilhelmstr. 27, Tübingen, 72070 Germany; 2 German Center for Infection Research (DZIF), Tübingen, Germany; 3 Institute fo rClinical Epidemiology and Applied Biometry, Faculty of Medicine, Eberhard Karls University Tübingen, Tübingen, Germany; 4 University of Heidelberg, Heidelberg, Germany; 5 Centre de Recherches Médicales de Lambaréné (CERMEL), Lambaréné, Gabon; 6 Leiden University Center for Infectious Diseases (LUCID), Leiden University Medical Center (LUMC), Leiden, The Netherlands; 7 Fondation pour la Recherche Scientifique (FORS), Cotonou, Benin; 8 General Pediatrics, Pediatric Neurology and Developmental Medicine, University Children’s Hospital Tübingen, Tübingen, Germany

**Keywords:** Malaria, Newborn, Neonate, Treatment

## Abstract

**Background:**

Malaria in neonates (age ≤ 28 days) remains under-recognized and poorly characterized, without specific management guidelines. We conducted a systematic review to collate available evidence on antimalarial treatment in this age group.

**Methods:**

We searched PubMed and Web of Science for studies reporting confirmed *Plasmodium spp.* infection and antimalarial treatment in neonates. Any type of study with original data, including case reports, was included if published in English, German or French. Demographic, clinical and antimalarial treatment data were extracted and summarized descriptively.

**Results:**

Out of 4028 reports identified, 26 publications (22 case reports, three case series, one cross-sectional study) reporting on antimalarial treatment in 40 neonates with confirmed malaria were included. The majority (75%) of publications originated from malaria-endemic regions with 19 cases reported from African countries. Median age at symptom onset and diagnosis was 16 and 19 days, respectively. *Plasmodium falciparum* and *P. vivax* accounted for 45% and 43% of cases, one case had a *P. falciparum* and *P. vivax* co-infection, and in four cases species of* Plasmodium * was not reported. All but one neonate were reported with symptomatic malaria, with fever, jaundice, and anaemia as predominating symptoms; 70% were initially misdiagnosed with sepsis. Treatment regimens varied substantially. Of neonates with *P. falciparum* malaria, only 28% received artemisinin-based therapy and most (56%) were treated with oral amodiaquine or chloroquine monotherapy. *P. vivax* malaria was mostly treated with oral chloroquine (64%). Oral drug administration practice was rarely reported but included drug administration as syrup or crushed tablets and use of an orogastric tube. All patients survived, with reported parasitaemia clearance in 2–7 days.

**Conclusion:**

The literature on newborn malaria management remains notably sparse. Reported management practices are highly inconsistent with regimens deviating from current treatment recommendations. Prospective interventional studies on the management of malaria in newborns, including evaluation of oral drug administration practices and pharmacokinetics are urgently needed to inform evidence-based age-specific guidelines.

**Clinical trial:**

Not applicable.

**Supplementary Information:**

The online version contains supplementary material available at 10.1186/s12879-025-12230-5.

## Introduction

Malaria remains one of the most significant global health challenges, with over 263 million cases and more than 597,000 deaths reported in 2023, most of which occur in children under five years of age [1]. While the burden of malaria in infants and young children is better documented, malaria in neonates (newborns within the first 28 days of life), remains poorly understood, under-reported, and often misdiagnosed [2].

Malaria in the first month of life can occur either via transplacental transmission during pregnancy or during delivery (congenital malaria), or through postnatal infectious mosquito bites or blood transfusions (acquired neonatal malaria). Congenital malaria is defined as clinical malaria under natural condition within the first seven days of life, as the prepatent period of mosquito-acquired malaria exceeds seven days, thereby allowing differentiation from postnatally acquired infection. Earlier literature and older studies concluded that malaria in newborns is uncommon and that neonatal malaria generally has limited clinical relevance for newborns, likely due to protective maternal antibodies, low parasite densities, foetal haemoglobin, and non-specific or absent symptoms in affected neonates [3]. However, maternally transferred antibodies and foetal haemoglobin do not provide universal protection against early-life malaria; their protective effects are limited, variable, and depend on specific circumstances such as exposure timing and duration, malaria antigen load, degree of placental inflammation, and presence of sickle cell disease [4–7]. Indeed, recent studies show that malaria in newborns is not uncommon [2, 8].

Malaria in neonates commonly presents with non-specific signs such as fever, poor feeding, lethargy, and jaundice, which overlap with sepsis and other neonatal illnesses [2]. This substantial symptom overlap can lead to missed or delayed malaria diagnoses if not actively considered. Moreover, guidelines for grading malaria severity and guiding clinicians in oral versus parenteral antimalarial treatment decisions are based on criteria for older children and adults and therefore of limited applicability to neonates.

In malaria-endemic settings, low-density parasitemia in neonates is probably underrecognized, with detection limited by routine diagnostics and nonspecific presentations; however, causally linking such low-level infections to clinical symptoms is difficult because maternally transferred antibodies, reduced vector exposure, and neonatal factors (e.g., foetal haemoglobin) collectively dampen parasite densities and clinical manifestation, in contrast to overt clinical malaria in newborns seen in non-immune, imported cases.

Specific evidence-based treatment guidelines for malaria in neonates are lacking at both national and international levels. Although the World Health Organization (WHO) offers general recommendations for malaria management in children under five years of age, these do not include tailored protocols for neonates [9]. Neonates are frequently excluded from clinical trials, resulting in a significant lack of age-specific pharmacokinetic and pharmacodynamic data for antimalarial drugs in this age group [10, 11]. In clinical practice, antimalarial regimens for neonates are often extrapolated from older paediatric populations or adjusted based on local clinical judgment and available drug formulations. This approach raises important concerns regarding dosing accuracy, treatment safety, and therapeutic efficacy, given the distinct physiologic and pharmacokinetic characteristics in neonates.

In view of the neglect of malaria in newborns and the lack of available evidence and standardized management protocols, we aimed to collate existing literature on the management of malaria in newborns through a systematic review.

## Methods

The primary objective of this systematic review was to collate the available literature on malaria management in neonates up to 28 days of age. Specifically, we aimed to review antimalarial treatment administration to neonates, including drugs, treatment regimens and dosages, as well as administration practices.

We systematically searched PubMed and Web of Science for relevant literature published since 1st January 2003 up to 30th April 2024. The start year of 2003 was chosen, as it marks the introduction of artemisinin-based combination therapy (ACT) in many endemic countries. Original studies, including case reports, were included if they were published in English, German, or French, involved neonates up to 28 days of age at treatment initiation with confirmed asexual *Plasmodium spp.* parasitaemia, and reported antimalarial treatment.

**Fig. 1:**
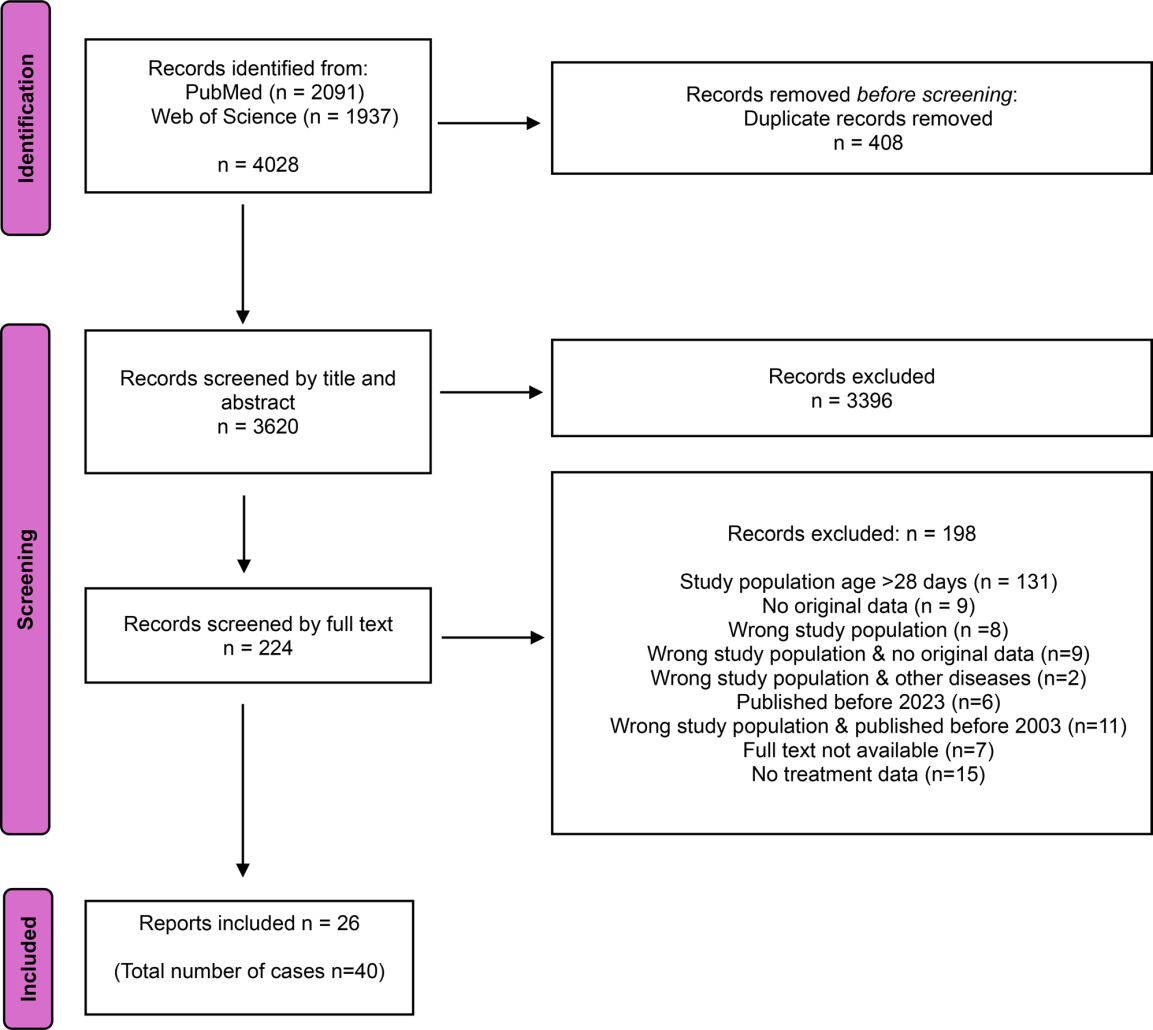
Flowchart of study selection process

Identified records were systematically screened using Rayyan software [12]. Three reviewers independently screened the records, resolving conflicts by consensus, with a fourth reviewer involved if necessary. Subsequently, all remaining records underwent independent title and abstract screening by all three reviewers, followed by full-text review of non-excluded records (Fig. 1). For data extraction, a pilot phase was carried out in which five records were double-extracted to confirm consistency among reviewers. After successful alignment was established, the remaining data were independently extracted by each reviewer using a standardized form in Covidence software (Veritas Health Innovation, Australia). Extracted demographic and clinical data included title, year of publication, study design, country or countries of data collection, age, gestational age at birth, sex, weight, height, history of symptoms onset, clinical presentation, diagnosis, history of migration, maternal age, gravidity, parity, and maternal history of malaria. Extracted diagnostic data comprised parasitology methods, malaria species, parasitaemia levels, as well as neonatal haemoglobin levels and platelet counts and concomitant diagnoses. Extracted data on malaria management encompassed drugs, route of administration, dosage, duration of treatment, as well as administration practices.

Data were summarized using descriptive statistics to provide an overview of key findings. Continuous variables were reported as medians with interquartile ranges (IQRs), while categorical variables were reported as frequencies and percentages. Due to limited availability of studies and heterogeneity in study design, populations, and reported treatment outcome determinants, an intended meta-analysis was not conducted. Visual aids, such as tables and figures, were used to present the data concisely and facilitate comparison across reports. Risk of bias was assessed using the Critical Appraisal Tools for Case Reports and the Critical Appraisal Checklist for Analytical Cross-Sectional Studies in JBI Systematic Reviews [13, 14]. Each domain was rated as Yes, No, Unclear, or Not Applicable. Risk of bias was assessed independently by two reviewers, and conflicts resolved by a third reviewer. We followed the PRISMA 2020 Guideline for reporting systematic reviews and meta-analysis. The protocol for the systematic review was registered on PROSPERO database (CRD42023414278).

## Results

Following the bibliographical search, 4,028 reports were identified. After removing duplicates and screening titles and abstracts, 224 publications were deemed possibly eligible, and their full texts were assessed for final inclusion (Fig. 1).

Ultimately, 26 publications encompassing 40 neonates reported between 2003 and 2024 were retained [15–40]. Of these, 22 were case reports reporting on single cases [15–18, 20–30, 32–35, 37, 39, 40], three were case series [19, 31, 36] reporting on a total of nine cases, and one was a cross-sectional study reporting on nine cases [38]. Following risk of bias assessment, 18 (72%) out of 25 case reports or case series were rated as of low risk of bias and 7 (28%) of moderate risk of bias; the cross-sectional study was rated as moderate risk of bias. Full grading is provided in Supplementary Tables 1 and 2.

**Fig. 2:**
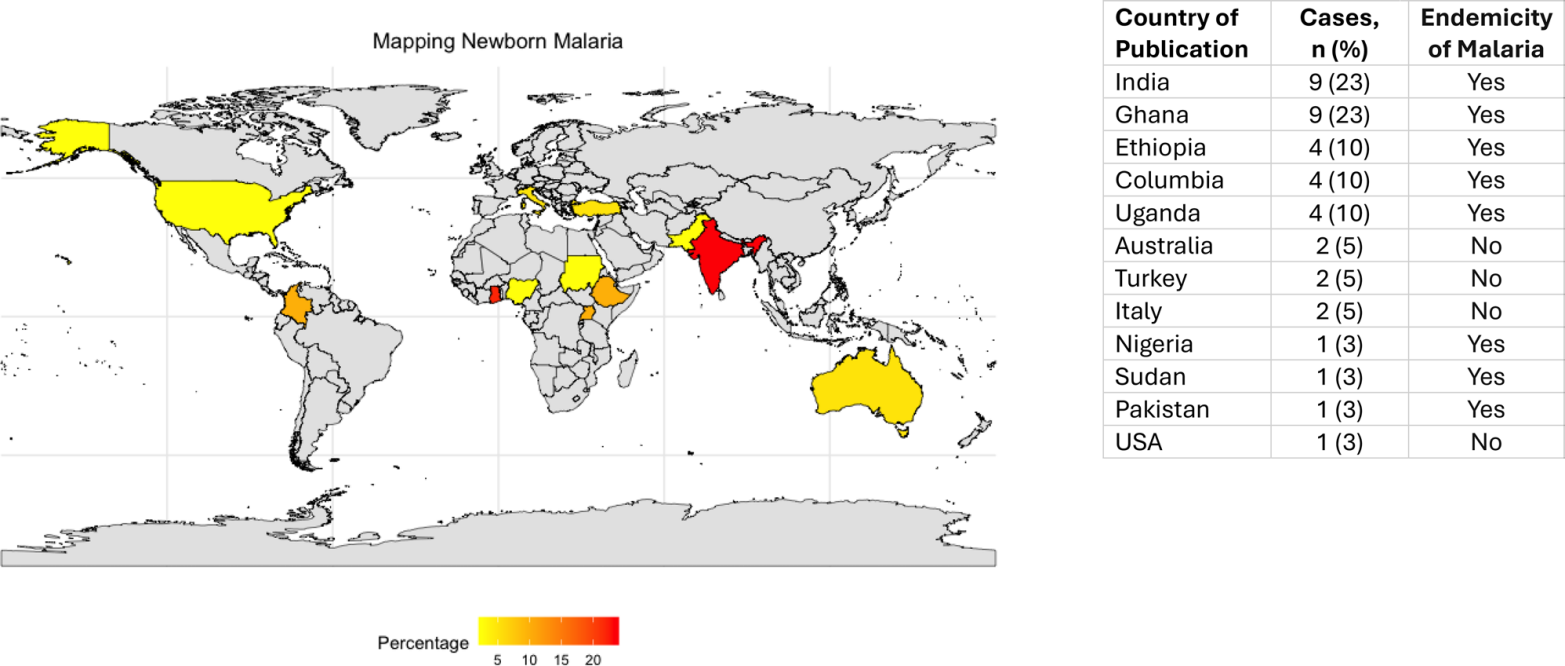
Distribution of cases per countries. World map showing the countries of origin of reported neonatal malaria cases included in this review

In this review, 30 out of 40 (75%) cases originated from malaria-endemic countries and 10 out of 40 (25%) from non-endemic countries; in three out of these 10 cases, the mother had migrated from a malaria endemic country (Fig. 2). One neonate acquired malaria by transfusion [34].

Of the 29 neonates with known sex, 15 (52%) were female. The median age at symptom onset and diagnosis was 16 and 19 days, respectively. All cases were parasitologically confirmed; microscopy was the primary diagnostic method (used in 98% (39 out of 40) cases), followed by rapid diagnostic tests (RDTs) (in 33% (13 out of 40) cases) and molecular methods (in 28% (11 out of 40) cases). Species were determined in 36 cases with *P. falciparum* detected in 18 (45%) cases, *P. vivax* in 17 (43%) cases, and *P. vivax / P. falciparum* co-infection in one case. Parasitaemia levels ranged from 83 to 26,700 parasites/µl for *P. falciparum* and 3,000 to 24,480 parasites/µl for *P. vivax*. Neonatal haemoglobin concentrations varied from 4.6 to 16.3 g/dL in *P. falciparum* infections and from 5.1 to 12.8 g/dL in *P. vivax* infections (Table 1).

**Table 1:** Demographic and parasitological characteristics by *Plasmodium* species

	Total	Species undetermined	*P*. *falciparum*	*P*. *vivax*	*P*. *vivax*/*P*. *falciparum*
n (%)	40 (100)	4 (10)	18 (45)	17 (43)	1 (3)
Female sex, n (%)	15/29 (52)	2 (7)	6 (21)	6 (21)	1 (3)
Age at symptoms onset, median days [IQR]*	16 (1–26)	-	17 (2–23)	16 (1–26)	25
Age at diagnosis, median days [IQR]**	19 (2–33)	-	18 (5–25)	20 (2–33)	25
Parasiteamia, parasites/µl, range (median)****	83-26700 (8341)	-	83–26,700 (5400)	3000–24,480 (10000)	-
Haemoglobin g/dl, median [IQR]***	10.5 (4.6–16.3)	-	11 (4.6–16.3)	11 (5.1–12.8)	8
Primigravidae, n (%)	10/13 (77)	-	3/13 (23)	7/13 (54)	-
Multigravidae, n (%)	3/13 (23)	-	2/13 (15)	1/13 (8)	-
Primiparity, n (%)	12/18 (66)	-	3/18 (17)	9/18 (50)	-
Multiparity, n (%)	6/18 (33)	-	5/18 (28)	1/18 (6)	-
Positive history of malaria in mothers, n (%)	15/23 (75)		5/9 (56)	10/14 (71)	

*total *n* = 20, **total *n* = 26, ***total *n* = 24, ****total *n* = 9

Most mothers were primigravidae and primiparous (Table 1). Among those mothers with available malaria history, three quarter had experienced malaria: one mother before pregnancy (*P. vivax*), seven mothers during pregnancy (*P. vivax* *n* = 5, *P. falciparum* *n* = 2), and seven mothers postpartum following diagnosis of malaria in their newborn (*P. vivax* *n* = 3, *P. falciparum* *n* = 4). Of the eight mothers with malaria prior or during pregnancy, six had received antimalarial treatment, including sulfadoxine-pyrimethamine, quinine, chloroquine, artemether-lumefantrine, or non-specified therapy.

Overall, 98% (39 out of 40) cases were symptomatic. Fever was the leading symptom in both *P. vivax* and *P. falciparum* cases, followed by jaundice, aneamia and pallor; reported symptoms are detailed in Table 2. Data did not allow for classification of uncomplicated versus complicated malaria in most cases. One child, reported as having cerebral malaria, had seizures, but the number of seizures within 24 h was not reported [17]. One child with unknown parasitaemia had severe anaemia with haemoglobin < 5 g/dl [17]. Seventy percent (28 out of 40) of neonates were initially misdiagnosed with conditions other than malaria, with sepsis being the most common misdiagnosis, reported in 61% (17 out of 28) of neonates.

**Table 2:** Symptoms per species

Symptoms	Total *N* = 36	*P*. *falciparum* *N* = 18	*P*.* vivax* *N* = 17	Mixed *P*.* vivax*/*P*. *falciparum* *N* = 1
Fever, n (%)	29 (81)	14 (78)	14 (82)	1
Jaundice, n (%)	17 (47)	12 (67)	4 (22)	1
Anaemia, n (%)	15 (42)	2 (11)	13 (76)	-
Pallor, n (%)	16 (44)	5 (28)	11 (61)	-
Splenomegaly, n (%)	15 (42)	4 (22)	10 (59)	1
Hepatomegaly, n (%)	12 (33)	3 (17)	8 (47)	1
Poor feeding, n (%)	12 (33)	5 (28)	6 (35)	1
Irritability, n (%)	6 (17)	3 (17)	3 (18)	-
Lethargy, n (%)	5 (14)	2 (11)	2 (12)	1
Dyspnoea, n (%)	4 (11)	2 (11)	2 (12)	-
Tachypnoea, n (%)	4 (11)	1 (6)	2 (12)	1
Vomiting, n (%)	4 (11)	2 (11)	2 (12)	-
Thrombocytopenia, n (%)	4 (11)	-	4 (23)	-
Cough, n (%)	3 (8)	1 (6)	2 (12)	-
Tachycardia, n (%)	2 (5)	-	2 (12)	-
Diarrhoea, n (%)	2 (5)	-	2 (12)	-
Abdominal distention, n (%)	2 (5)	-	2 (12)	-
Weight loss, n (%)	1 (3)	-	1 (6)	-
Respiratory distress syndrome, n (%)	1 (3)	-	1 (6)	-
Blood in stool, n (%)	1 (3)	-	1 (6)	-
Seizure, n (%)	1 (3)	1 (6)	-	-
Constipation, n (%)	1 (3)	1 (6)	-	-

One neonate with *P. falciparum* infection diagnosed at the age of 5 days was asymptomatic [40]; the mother reported a possible malaria infection at 32 weeks of pregnancy, treated non-specifically. Based on known maternal malaria during pregnancy, the baby was closely monitored postnatally and *P. falciparum* trophozoites with 1.5% parasitaemia were detected five days after birth.

Treatment regimens varied considerably between reports (Table 3). Among the 18 *P. falciparum* infection cases only 5 (28%) cases received artemisinin-based therapy. The majority of newborns with *P. falciparum *infection (10 out of 18 (56%)) were treated with oral antimalarial medication alone. Three neonates received combined parenteral and oral treatment, and five neonates were given parenteral treatment only.

**Table 3:** Antimalarial treatment regimens and administration practices

	*n*	Parenteral administration	Parenteral and oral administration	Oral administration	Paediatric Formulations
***P. vivax (n = 17)***					
Chloroquine	10			*N* = 7; 3 days (0, 6, 24,48 h), initial dose 10 mg/kg, followed by 5 mg/kg [8, 11, 12, 14, 16, 18, 21, 22, 23, 25, 28, 32, 37] *N* = 3; 3 days, total dose 25 mg/kg [13, 18, 22, 24, 29, 33]	1 syrup [7, 18] 1 orogastric tube [14, 25]
Chloroquine Quinine	2		*N*= 1; CQ (oral) 3 days, initial dose 10 mg/kg, followed by 5 mg/kg; followed by Q (iv), dosage and duration unknown [23, 34] *N* = 1; CQ (oral) 2 days, total dose 25 mg/kg; Q (iv) loading dose 20 mg/kg, followed by 10 mg/kg 8-hourly [9, 20]		NR
Artesunate	2	*N* = 1; AS 5 days, 4 mg/kg [19, 21]		*N* = 1; AS 7 days, 4 mg/kg day 1, 2 mg/kg day 2–7 [35]	NA
Artesunate Clindamycin	2	*N* = 1; AS 7 days, 2.4 mg/kg; C 7 days, 20 mg/kg/d [19, 21] *N*= 1; AS 4 days, 2.4 mg/kg; C 7 days, loading dose 10 mg/kg, followed 8-hourly by 5 mg [16, 5]			NA
Chloroquine Primaquine	1			*N* = 1; CQ (2days), initial dose 10 mg/kg, followed by 5 mg/kg; PQ 14 days, 0.3 mg/kg/d [15]	NR
***P. falciparum (n = 18)***					
Amodiaquine	9			*N*= 9; Amodiaquine 10 mg/kg/d oral (duration unknown) [38]	NR
Artesunate	3	*N* = 3; AS 7days, 3 mg/kg/d [25, 36]			NA
Quinine	2	*N* = 1; 3 days 10 mg/kg 8-hourly, [8^8^, ^19^] *N* = 1; 3 days, initial 20 mg/kg followed by 10 mg/kg 8-hourly [8, 19]			NA
Quinine, Clindamycin	1		*N* = 1; day 1 Q loading dose then 10 mg/kg + C 5 mg/kg iv every 8 h Day 2 switch to oral Q (10 mg/kg) + C (20 mg/kg/day) - total duration of treatment 7d [16, 27]		NA/NR
Artesunate, DHP	1		*N* = 1; AS (iv) 2 days (0,12,24 h), 3.4 mg/kg/d; DHP (oral) 3 days, 2/16 mg/kg/d [39, 28]		Crushed 39 [28]
Chloroquine	1			*N* = 1; CQ 10 mg/kg 2 days, 5 mg/kg 1 day [29]	NR
Artesunate, Artesunate- Amodiaquine	1		*N* = 1; AS (iv) 3 days, 3 mg/kg; followed by ASAQ (oral) 4 and 10 mg/kg/d [6, 17]		NR
***P. vivax/P. falciparum (n = 1)***					
Artesunate, Chloroquine, Quinine	1		*N* = 1; AS iv (dosage and duration unclear); CQ syrup (dosage and duration unclear), Q iv (standard dose, duration unclear); drugs consecutively 26 [15]		Syrup [15, 26]

CQ = Chloroquine; Q = Quinine; AS = Artesunate; C = Clindamycin; PQ = Primaquine; DHP = Dihydroartemisinin-Piperaquine; ASAQ = Artesunate-Amodiaquine; NR=not reported

For *P. vivax* infections, oral chloroquine was the most frequently used treatment in 13 out of 17 (76%) cases. The typical chloroquine regimen consisted of a 10 mg/kg loading dose followed by 5 mg/kg daily for three days. In two cases oral chloroquine was combined with intravenous quinine and in one case with primaquine. Alternative *P. vivax* treatments included intravenous artesunate administered over four to seven days, either alone or combined with parenteral clindamycin.

Concomitant sepsis treatment involved intravenous antibiotics such as cefotaxime and amikacin.

Parasite clearance following antimalarial treatment (reported in 19 cases) ranged from two to seven days. All patients survived to discharge and until last reported follow-up, which ranged between one week to one year post-discharge.

## Discussion

This review identified 40 cases of neonatal malaria from 26 publications being published between 2003 and 2024 and reporting on antimalarial treatment. Most of the studies were single case reports, with only a few small case series and one cross-sectional study. This predominance of anecdotal reporting underscores the lack of systematic research and an ongoing critical evidence gap in management of neonatal malaria over the past two decades. Most cases were reported from endemic countries; however, a fifth of cases were identified in non-endemic settings, with several neonates born to mothers who had migrated from endemic regions. These findings emphasize the global relevance of neonatal malaria and the importance of clinical vigilance even in non-endemic regions.

This review reveals significant heterogeneity in the treatment of neonatal malaria, particularly for *P. falciparum* infections. Only one out of four (28%) neonates received an artemisinin-based regimen and over half of *P. falciparum* cases received oral monotherapy, despite WHO recommendations advocating ACTs as the first-line therapy. While all cases in this review survived, monotherapy generally carries increased riks for resistance emergence and treatment failure compared to combination regimes.

For *P. vivax* infections, chloroquine was the predominant treatment, administered in over two-thirds of cases. While this aligns with current WHO guidance for regions without documented resistance [9], the combination of chloroquine with intravenous quinine or primaquine in a few cases suggests diagnostic uncertainty or concern for resistance or relapse. Notably, few cases reported the use of primaquine, which is normally required to prevent *P. vivax* relapse by eliminating dormant liver forms (hypnozoites). However, primaquine is not needed in congenital malaria cases because the parasite is transmitted directly through the placenta to the bloodstage, bypassing the liver stage entirely.

Effective administration of oral antimalarial treatment is crucial for favourable treatment outcomes; however, oral drug administration is particularly challenging in newborns and young children. Paediatric formulations of antimalarials, particularly ACTs, have been developed for several compounds to ease oral treatment administration in young children and were found to have a beneficial tolerability profile [41]; however, their value was not evaluated in newborns. An important aspect of this review was therefore to assess the mode of oral drug administration in neonates, but oral drug administration practices were rarely reported.

Only recently the first optimized paediatric formulation of artemether-lumefantrine specifically designed for infants weighing less than 5 kg has been developed and evaluated in the CALINA study (NCT04300309). This formulation consists of dispersible tablets containing 2.5 mg artemether and 30 mg lumefantrine. The study demonstrated that an optimized formulation of 5 mg artemether and 60 mg lumefantrine twice daily in infants >2 to < 5 kg body weight resulted in safe and efficacious drug concentration ranges comparable to older paediatric patients [42]. Following positive results from the CALINA study, this formulation received regulatory approval from Swissmedic in July 2025 as Riamet Baby (Coartem Baby), becoming the first malaria treatment specifically approved for infants weighing 2–5 kg. Rapid approval is expected in selected African countries first, with the manufacturer planning to make the treatment available on a largely not-for-profit basis in malaria-endemic regions [43]. The CALINA study, including infants and neonates, will also provide some insight into drug administration in newborns weighing less than 5 kg. However, the complete clinical results regarding detailed administration practices and tolerability data are not yet publicly available.

Encouragingly, despite the diverse treatment regimens employed, all patients in this review survived, and parasite clearance occurred within 2–7 days in reported cases; there might, however, be reporting bias. While this suggests favourable short-term outcomes, the long-term effects of neonatal malaria and its treatment, particularly neurological sequelae or haematological recovery, remain poorly studied and warrant further investigation.

The clinical assessment and management of neonatal malaria is complicated by the nonspecific nature of symptoms, which frequently overlap with other conditions such as sepsis [8], as also reflected in this review. This diagnostic complexity is compounded by the fact that established WHO criteria for distinguishing between uncomplicated and severe malaria, developed primarily for older children and adults, may not be directly applicable or appropriate for neonatal patients. In line with this challenge, none of the included articles in this review classified the neonatal cases as either uncomplicated or severe malaria. Consequently, clinical guidelines that recommend oral versus parenteral antimalarial treatment based on disease severity classifications become challenging to implement in neonates, where standard severity markers may be absent or unreliable. In settings where appropriately formulated oral antimalarial drugs for neonates are unavailable, healthcare providers may justifiably prefer intravenous treatment to ensure adequate drug exposure and therapeutic efficacy, also in cases that do not meet severe malaria criteria. However, this preference for parenteral therapy may create challenges in low- and middle-income countries, where multiple interconnected barriers can limit safe and effective intravenous treatment. These challenges include under-resourced medical infrastructure, such as insufficient neonatal care units, shortage of trained personnel for neonatal iv line insertion and monitoring, and insufficient supply resources for appropriate-sized iv cannulas and precise dosing preparations of low doses. This infrastructure gap underscores the urgent need for suitable oral formulations specifically designed for neonatal use. Furthermore, current treatment guidelines recommend transitioning from parenteral to oral therapy once clinical improvement is observed, meaning that even neonates who initially receive intravenous treatment will ultimately require appropriate oral formulations to complete their antimalarial course. This dual requirement of appropriate oral antimalarials for neonates, for both effective completion of initial parenteral management in severe malaria and oral treatment in uncomplicated malaria, highlights the critical importance of developing age-appropriate antimalarial drugs for this vulnerable population.

This systematic review has several limitations. Overall, the inability to conduct the originally planned meta-analysis due to limited study availability and substantial heterogeneity in study designs, populations, and outcome measures represents a significant limitation that impacts the strength and generalizability of our findings. Data are predominantly derived from case reports and small case series, which inherently introduce reporting and publication bias. The heterogeneity in diagnostic approaches and treatment regimens complicates the assessment of efficacy and clinical decision-making. The lack of standardized definitions for neonatal malaria severity, especially in distinguishing between uncomplicated and severe forms, further restricts clinical interpretation of appropriate management practices. Further, language restriction for literature search may have excluded further reports. Finally, due to the small number of reported cases the results may not fully capture the diversity of neonatal malaria presentations and management across different geographic and healthcare contexts.

## Conclusion

This systematic review highlights wide inconsistencies in management of newborn malaria, particularly for *P. falciparum* infections, where the use of non-recommended therapies was common. Applicability of malaria severity criteria guiding intravenous versus oral treatment in older children should be evaluated for newborns taking into account possible metabolic particularities in this population. Future research should focus on pharmacological studies tailored to newborns, the inclusion of this age group in antimalarial drug trials, including evaluation of best strategies for oral versus parenteral treatment administration in newborns.

## Electronic supplementary material

Below is the link to the electronic supplementary material.

Supplementary Material 1

## Data Availability

Data available upon request to the corresponding author.
