# Supplementary material for: Management of malaria in newborns: a systematic review
Source: BMC Infect Dis. 2025 Dec 5;25:1705. doi: 10.1186/s12879-025-12230-5 (PMC12690788; doi:10.1186/s12879-025-12230-5)
Supplement: Supplementary file 1 — Supplementary Material 1 [file 12879_2025_12230_MOESM1_ESM.docx]

**Supplementary Material:**

Supplementary Table 1: JBI Critical Appraisal Checklist Case Report

| Author | Q1 | Q2 | Q3 | Q4 | Q5 | Q6 | Q7 | Risk of Bias |
| --- | --- | --- | --- | --- | --- | --- | --- | --- |
| Nwaneli 2022 |  |  |  |  |  |  |  | Low |
| OrdoñezDíaz 2021 |  |  |  |  |  |  |  | Moderate |
| Gopikrishnan 2021 |  |  |  |  |  |  |  | Moderate |
| DelCastilloCalderón 2020 |  |  |  |  |  |  |  | Low |
| Saghir 2020 |  |  |  |  |  |  |  | Moderate |
| GebremeskelTekle 2018 |  |  |  |  |  |  |  | Low |
| DelCastillo 2017 |  |  |  |  |  |  |  | Low |
| Gulasi 2016 |  |  |  |  |  |  |  | Low |
| Olupot-Olupot 2018 |  |  |  |  |  |  |  | Moderate |
| Shah 2015 |  |  |  |  |  |  |  | Moderate |
| Prashanth 2012 |  |  |  |  |  |  |  | Low |
| Orostegui-Pinilla 2011 |  |  |  |  |  |  |  | Low |
| Chandelia 2013 |  |  |  |  |  |  |  | Moderate |
| Hi 2011 |  |  |  |  |  |  |  | Low |
| Sankar 2010 |  |  |  |  |  |  |  | Moderate |
| Mohan 2010 |  |  |  |  |  |  |  | Low |
| Poespoprodjo 2010 |  |  |  |  |  |  |  | Low |
| DelPunta 2010 |  |  |  |  |  |  |  | Low |
| Avabratha 2010 |  |  |  |  |  |  |  | Low |
| Valecha 2007 |  |  |  |  |  |  |  | Low |
| Baspinar 2006 |  |  |  |  |  |  |  | Low |
| Hewson 2003 |  |  |  |  |  |  |  | Low |
| Virdi 2003 |  |  |  |  |  |  |  | Low |
| Zerubabel Girma Tesso 2024 |  |  |  |  |  |  |  | Low |
| Misganu Teshoma Regasa 2024 |  |  |  |  |  |  |  | Low |

Q1. Were patient’s demographic characteristics clearly described?

Q2. Was the patient’s history clearly described and presented as a timeline?

Q3. Was the current clinical condition of the patient on presentation clearly described?

Q4. Were diagnostic tests or assessment methods and the results clearly described?

Q5. Was the intervention(s) or treatment procedure(s) clearly described?

Q6. Was the post-intervetion clinical conditon clearly described?

Q7. Does the case report provide takeaway lessons?

Yes, No, Unclear

Supplementary Table 2: JBI Critical Appraisal Checklist for Analytical Cross-Sectional Studies

| Author | Q1 | Q2 | Q3 | Q4 | Q5 | Q6 | Q7 | Q8 | Risk of Bias |
| --- | --- | --- | --- | --- | --- | --- | --- | --- | --- |
| Enweronu-Laryea 2013 |  |  |  |  |  |  |  |  | Moderate |

Q1. Were the criteria for inclusion in the sample clearly defined?

Q2. Were the study subjects and the setting described in detail?

Q3. Was the exposure measured in a valid and reliable way?

Q4. Were objective, standard criteria used for measurement of the condition?

Q5. Were confounding factors identified?

Q6. Were strategies to deal with confounding factors stated?

Q7. Were the outcomes measured in a valid and reliable way?

Q8. Was appropriate statistical analysis used?

Yes, No, Unclear, Not Applicable
